# FDG PET-CT demonstration of metastatic neuroendocrine tumor of prostate

**DOI:** 10.1186/1477-7819-6-64

**Published:** 2008-06-19

**Authors:** Yiyan Liu

**Affiliations:** 1 Nuclear Medicine Service, Department of Radiology, University Hospital, UMDNJ, Newark, New Jersey, USA

## Abstract

**Background:**

FDG PET-CT is generally not suitable for diagnosing prostate cancer because of low glycolysis of the tumor cells. Neuroendocrine differentiation of the prostate cancer is often associated with early visceral metastasis and dismal prognosis, which is resulted from changed metabolic and regulatory pathways.

**Case presentation:**

A case is reported in this paper that FDG PET-CT demonstrates intense uptake of neuroendocrine tumor of the prostate and multiple metastases.

**Conclusion:**

There is high glycolysis and strong FDG-avidity of neuroendocrine tumor of the prostate, which is similar to that of high grade of neuroendocrine tumor in other tissue and organs. In some selected cases of prostate neuroendocrine cancer, whole body FDG PET-CT may be helpful for detection of metastatic disease.

## Background

Positron emission tomography (PET) is a new imaging modality which has been widely used for detection of metastasis in various malignancies. The most used radiotracer, F18-fluorodeoxyglucose (FDG) is for evaluation of glycolysis and glucose transporter expression. It is well known that most of malignant tumors display increased glucose metabolism. Unfortunately FDG-PET has not been very helpful in prostate cancer because of low glycolysis of the tumor cells. In addition, physiologic urinary excretion of FDG can interfere with imaging of the pelvis [1-3].

Neuroendocrine differentiation of the prostate cancer contributes to the progression of the disease and often associated with visceral metastases and dismal prognosis [4,5]. We herein describe the case of a prostate cancer that had neuroendocrine differentiation and multiple metastatic lesions detected by FDG PET-CT.

## Case presentation

A 79 year old male with a history of prostate cancer was treated with external radiation and hormone a few years ago. He recently developed gross hematuria and renal failure. CT scan showed bladder mass, hydronephrosis and small lung nodules, but no liver lesion or retroperitoneal lymphadenopathy was noted. The patient underwent nephrostomies and prostate biopsy.

Immunohistochemical studies of the prostate tissue revealed positive staining of the tumor cells for chromogranin, synaptophysin and neuro-specific enolase. The samples were negative for cytokeratin and PSA. In view of the patient's history of prostate adenocarcinoma, the findings were consistent with a high grade of neuroendocrine differentiation.

**Figure 1:**
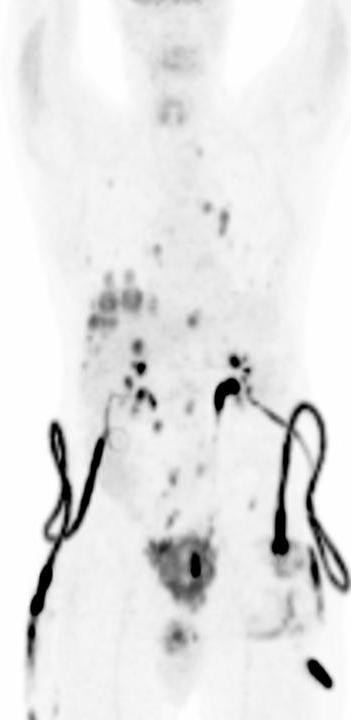
Maximum intensity projection (MIP) of the whole body FDG PET-CT imaging. There is intense uptake in the prostate tumor with invasion and infiltration to the bladder wall. Multiple FDG-avid metastatic lesions are noted in the liver, Lungs and lymph nodes of the mediastinum and retroperitoneum.

**Figure 2:**
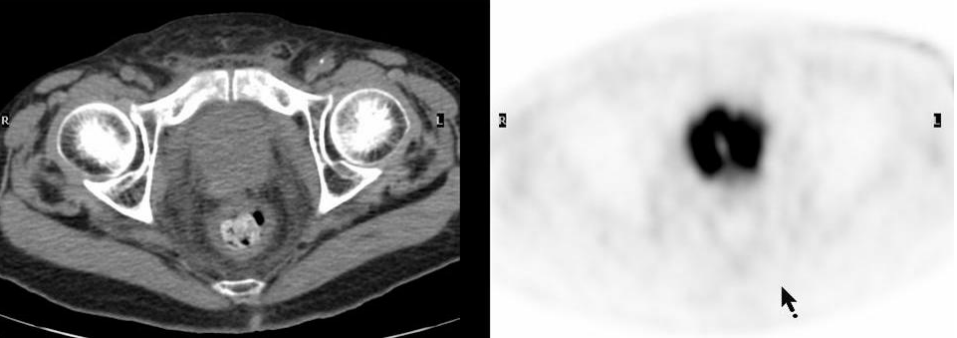
Neuroendocrine differentiation of the prostate cancer displays high grade of FDG uptake on transaxial PET-CT images, which is different from that noted in most of adenocarcinoma of the prostate.

**Figure 3:**
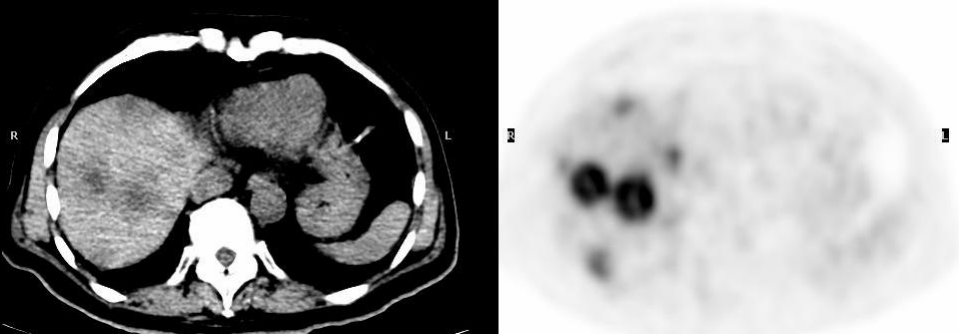
FDG PET-CT images demonstrate multiple necrotic hepatic metastases while bone scan and CT two months earlier were negative.

**Figure 4:**
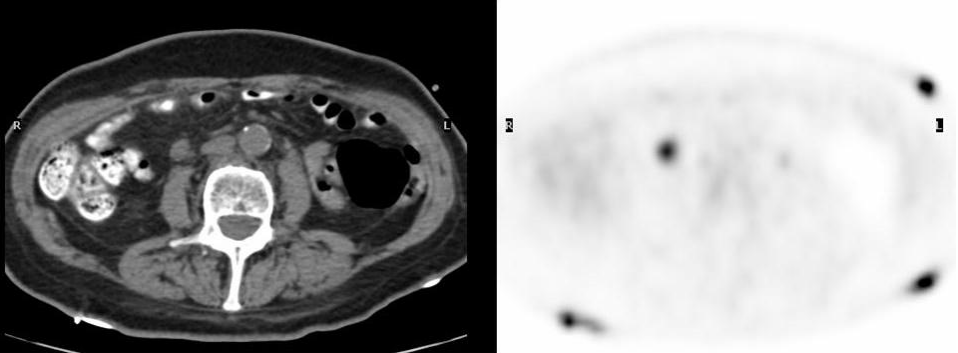
FDG PET-CT images demonstrate a 1.3 cm aortocaval lymph node with intense uptake consistent with metastatic disease.

The patient's whole body bone scintigraphy was negative. The medical oncologist recommended, the patient was also strongly interested in cystoprostatectomy with ileal conduit followed by chemotherapy. As a final pre-surgical work-up, the patient had FDG PET/CT, which demonstrated tumor invasion and infiltration to the bladder as well as multiple metastastic lesions in the liver, lungs and lymph nodes (Figure 1, 2, 3, 4). Compared to a CT less than two months earlier, PET-CT findings suggested marked progression of prostate tumor and multiple new metastases. Therefore, scheduled surgery was cancelled and the patient was treated with chemotherapy (Topotecan) in a hospice facility.

## Discussion

Neuroendocrine tumor of the prostate is featured by early visceral rather than bone metastases [6]. In this case, a CT scan two months earlier was negative in the liver and for lymphadenopathy, but FDG PET-CT demonstrated extensive hepatic lesions as well as nodal and lung metastases while bone scan was still negative.

FDG-PET scan has its advantages of metabolic and molecular imaging, therefore early detection of malignant disease. In addition, routine whole body imaging make it best imaging modality for staging and restaging of most of the malignant diseases. In prostate adenocarcinoma, FDG-PET often does not display increased uptake. Liu et al found only 4% sensitivity for detecting primary prostate cancer with FDG-PET [7]. But using continuous bladder irrigation, Oyama et al found an increased sensitivity for detecting the prostate tumor [8]. Patients showing increasing prostate specific antigen (PSA) after definite local therapy for prostate cancer represent a diagnostic dilemma. FDG-PET may identify local recurrence and distant metastases with increasing PSA [9]. In addition, Morris et al reported that using PSA levels, bone scintigraphy and soft tissue imaging as references, FDG-PET might be a promising outcome measure after chemotherapy in prostate cancer [10].

With neuroendocrine differentiation, the tumor has different biological behavior [4]. The cells involved in this process secrete a variety of factors that can influence growth patterns and metabolic pathways [11]. This case suggests very high glycolysis and strong FDG-avidity of neuroendocrine tumor of the prostate, which is similar to that of high grade of neuroendocrine tumor in other tissue and organs [12].

To our knowledge, this is the first report of FDG-PET application in neuroendocrine tumor of the prostate and metastatic disease. In some selected cases of neuroendocrine tumor of the prostate, whole body FDG-PET may be helpful for detection of metastasis and therefore change patient's management.

## Conclusion

Neuroendocrine tumor of the prostate demonstrates different metabolic characteristics from adenocarcinoma. It has high FDG avidity and often demonstrates early visceral metastases. In some selected cases, FDG-PET may be an ideal imaging modality and helpful for identifying metastatic disease.

## Competing interests

The author declares that he has no competing interests.

## Authors' contributions

YL conceived and designed the study, and prepared the draft and final manuscript.
